# SIRT3 regulates mitochondrial biogenesis in aging-related diseases

**DOI:** 10.7555/JBR.36.20220078

**Published:** 2022-06-28

**Authors:** Hongyan Li, Zhiyou Cai

**Affiliations:** 1 Department of Neurology, the Affiliated Hospital of Southwest Medical University, Luzhou, Sichuan 646000, China; 2 Department of Neurology, Chongqing General Hospital, Chongqing 400013, China

**Keywords:** SIRT3, PGC1α, mitochondrial biogenesis, aging-related diseases

## Abstract

Sirtuin 3 (SIRT3), the main family member of mitochondrial deacetylase, targets the majority of substrates controlling mitochondrial biogenesis *via* lysine deacetylation and modulates important cellular functions such as energy metabolism, reactive oxygen species production and clearance, oxidative stress, and aging. Deletion of *SIRT3* has a deleterious effect on mitochondrial biogenesis, thus leading to the defect in mitochondrial function and insufficient ATP production. Imbalance of mitochondrial dynamics leads to excessive mitochondrial biogenesis, dampening mitochondrial function. Mitochondrial dysfunction plays an important role in several diseases related to aging, such as cardiovascular disease, cancer and neurodegenerative diseases. Peroxisome proliferator-activated receptor gamma coactivator 1-alpha (PGC1α) launches mitochondrial biogenesis through activating nuclear respiratory factors. These factors act on genes, transcribing and translating mitochondrial DNA to generate new mitochondria. PGC1α builds a bridge between SIRT3 and mitochondrial biogenesis. This review described the involvement of SIRT3 and mitochondrial dynamics, particularly mitochondrial biogenesis in aging-related diseases, and further illustrated the role of the signaling events between SIRT3 and mitochondrial biogenesis in the pathological process of aging-related diseases.

## Introduction

A research based on population showed that the aging population is gradually increasing throughout the world^[1–2]^. However, there is a general physical and functioning decline with aging. Aging is an independent risk factor for diseases. Most common aging-related diseases include Parkinson's disease (PD), Alzheimer's disease (AD), type 2 diabetes mellitus (T2DM), cardiovascular disease, and cancer^[2–3]^.

Mitochondria, a major source of energy, are important organelles to fuel the human body's functions. Thereby, mitochondrial dysfunction is viewed as a potential regulator of the aging process^[4]^. Although plenty of factors, including abnormal mitochondrial quality control and aging^[4]^, contribute to mitochondrial dysfunction, our attention is paid to mitochondrial biogenesis, yielding new mitochondria in response to various stress. Besides, mitochondrial dynamics will be discussed briefly.

Mitochondrial function is modulated by a series of enzymes, the majority of which are also deacetylated by sirtuin 3 (SIRT3). SIRT3 targets many substrates controlling mitochondrial biogenesis through lysine deacetylation and modulates important cellular functions such as mitochondrial sugar, fat, and amino acid metabolism as well as reactive oxygen species (ROS) production and clearance^[5]^.

This review summarizes recent research on mitochondrial biogenesis and SIRT3, further illustrating the effects of SIRT3 on mitochondrial biogenesis in aging-related diseases.

## SIRT3

Sirtuins are a family of NAD^+^‐dependent histone deacetylases/mono-ADP-ribosyl transferase enzymes (SIRT1–SIRT7) and highly conserved in both bacteria and humans during the evolution^[6–7]^. Sirtuins have been reported to regulate a wide variety of biological processes such as metabolism, mitochondria homeostasis, genomic stability, DNA repair, ROS homeostasis, and aging^[8–9]^. SIRT3 is primarily localized in the mitochondria and functions as a major mitochondrial deacetylase that targets a growing number of substrates involved in metabolic homeostasis, mitochondrial dynamics, mitochondrial unfolded protein response, and oxidative stress^[8]^. In addition, SIRT3 protein is highly expressed in the brain, heart, liver, and brown adipose tissue^[10]^. SIRT3 controls global mitochondrial lysine acetylation level, and the sum of acetyl modifications are critical for these biological processes^[10]^. For instance, SIRT3 deacetylates numerous mitochondrial metabolic enzymes such as isocitrate dehydrogenase and malate dehydrogenase in the tricarboxylic acid cycle^[11]^, complex Ⅰ, complex Ⅱ, and complex Ⅲ in the electron transport chain^[12]^, and long-chain acyl-CoA dehydrogenase involved in the fatty acid oxidation pathway^[13]^. SIRT3 regulates these biosynthetic pathways involved in glucose and lipid metabolism and provides adequate energy to cells or tissues. SIRT3 deacetylates enzymes related to cellular anti-oxidative defense capacity, like superoxide dismutase 2 (SOD2) associated with ROS homeostasis^[14]^, to prevent excessive ROS accumulation. SIRT3 can also stimulate the peroxisome proliferator-activated receptor gamma coactivator 1-alpha (PGC1α) controlling mitochondrial biogenesis *via* adenosine 5′-monophosphate (AMP)-activated protein kinase (AMPK) pathway to maintain mitochondrial quality and quantity^[15]^. Accordingly, SIRT3 is essential for sustaining anti-oxidative system, mitochondrial integrity, and normal mitochondrial function as the basis of physiological processes.

It is well known that SIRT3 is strongly involved in the aging process. Subsequent genetic studies found the relationship between single nucleotide polymorphisms (SNPs) of the *SIRT3* gene and longevity^[16–17]^. Three types of SNPs occurring in SIRT3 (G477T, variable number tandem repeat and V208I) have been discovered in human and SIRT3-knockout mice, and they can modify human health and lifespan^[16,18–19]^.

Upregulated SIRT3 increases energy generation to meet high energy demand during calorie restriction (CR), fasting, and exercise^[17]^. Accumulating evidence confirms that SIRT3 attenuation or ablation accelerates the development of aging-related diseases, including cancers, metabolic syndromes, cardiovascular diseases, and neurodegenerative diseases^[12,20–21]^. For example, SIRT3 protects dopaminergic neurons from degeneration and necrosis by regulating mitochondrial quality control, reducing mitochondrial oxidative stress, and downregulating α-synuclein (α-syn) in PD^[21–24]^. FOXO3 deacetylated by SIRT3 can maintain a balance between mitochondrial fission/fusion by inducing a series of FOXO3-dependent genes expression^[25]^. This process is beneficial in delaying the progression of PD. In response to stress situations, Ku70 is deacetylated by SIRT3, then binds to Bax, which protects cardiomyocyte against apoptosis induced by Bax^[26]^. SIRT3 was indicated to inhibit p53 activity, rescuing cells from apoptosis^[27]^. In PTEN-deficient cells, p53 at lysines 320 and 382 is modified by SIRT3, deteriorating the condition of non-small cell lung cancer^[28]^. Conversely, overexpression of SIRT3 in human glioma cells can promote tumor progression through Ku70-BAX interaction^[29]^.

## Mitochondrial biogenesis

**Figure 1:**
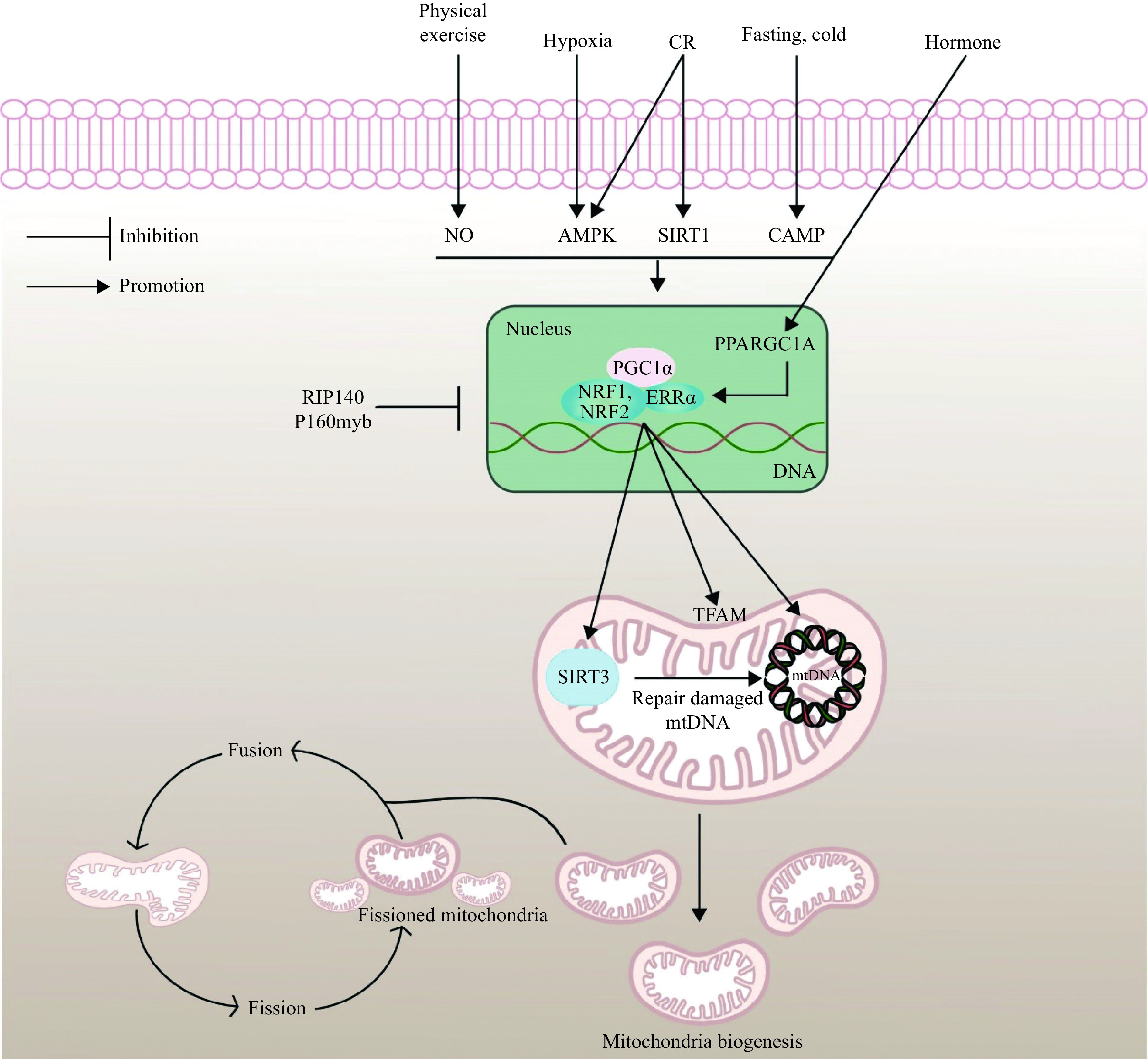
Regulators of mitochondrial biogenesis.

Mitochondrial biogenesis is a complex process of mitochondrial growth and division. It requires the synthesis of proteins encoded by nuclear and mitochondrial (mt) DNA, besides the biogenesis of new organellar structures^[30–31]^. The nuclear genome encodes enormous mitochondrial enzymes and proteins, thus regulating transcriptional networks^[32]^. The mitochondrial DNA (mtDNA) is responsible for essential components of the electron transport chain as well as all rRNAs and tRNAs^[33–34]^. Biological processes, including transcription and replication from both nuclear and mtDNA, must be coordinated with each other to generate new mitochondrion in response to high-demand for energy^[35]^ (***Fig. 1***).

It is well known that PGC1α is a primary regulator of mitochondrial biogenesis through activating nuclear respiratory factors (NRFs) and nuclear receptor subfamily (*e.g.*, estrogen-related receptor alpha [ERRα]), expressing in tissues and organs with high-energy-requirements^[35–36]^. Because they regulate the expression of mitochondrial respiratory subunits and mitochondrial transcription factors including mitochondrial transcription factor A (TFAM) and mitochondrial transcription factor B (mtTFB) isoform genes^[37]^. These genes drive the transcription and replication of mtDNA. PGC1α drives multiple transcription factors (NRFs, ERRα,* etc.*), thus creating new mitochondria in response to various stress conditions^[38]^.

The regulation of mitochondrial biogenesis is affected by multiple internal and external factors through PGC1α^[39]^. Because PGC1α, one member of the transcriptional coactivators family, controls various aspects of mitochondrial biogenesis, including initiation of respiratory chain and fatty acid oxidation genes, an increase of mitochondrial number, and augmentation of mitochondrial respiratory capacity^[33]^. At the level of molecule, factors involved in activation of PGC1α include nitric oxide, AMPK, CREB, SIRT1, NRF-1and NRF-2^[39–40]^. However, RIP140 and p160 myb binding protein take part in the inhibition of PGC1α and thus suppress mitochondrial biogenesis^[39]^. At the level of the organism, hormones also influence mitochondrial biogenesis. Under oxidative stress, the thyroid hormone triiodothyronine can drive mitochondrial biogenesis and respiration process in various tissues of mouse^[41–43]^, and steroid hormones stimulate production of mitochondrial proteome^[39]^. Hormones are capable of controlling mitochondrial biogenesis by affecting PPARGC1A level^[38]^. Besides, physiological environment changes are also reported to modulate mitochondrial biogenesis such as exercise, CR, hypoxia, stress, and temperature^[40]^, and such observations were shown in rats and humans^[44–45]^.

In addition to mitochondrial biogenesis, mitochondrial fission (mito-fission) is a process in which mitochondria are broken up into smaller fragments, thereby producing two daughter mitochondria to increase mitochondrial mass during cell self-renewal^[46]^. During mito-fission, the damaged mitochondrion is removed by mitophagy, whereas the healthy one steps into the next phase, which is mitochondrial biogenesis^[47]^. Mito-fission is launched by endoplasmic reticulum (ER). Because once ER contacts with mitochondria, the sites at which dynamin-related protein 1 (DRP1) is recruited and assembles will be established^[48]^. DRP1, cutting the inner and outer membranes, also recruits some proteins aiding in splitting into two segments. Those proteins consist of mitochondrial receptor protein 1 (Fis1), mitochondrial fission factor (Mff), and mitochondrial dynamic proteins (MIDs)^[47,49–50]^. When cells are exposed to stresses, mito-fission bears the responsibility to maintain mitochondrial network functioning properly and promotes mitochondrial trafficking through segregating defective mitochondria and preserving the normal one^[50]^.

Facilitating mitochondrial biogenesis would be advantageous in many disease models, but once the balance of mitochondrial dynamics (mito-fission, mitophagy, and mitochondrial biogenesis) is destroyed, excessive mitochondrial biogenesis is detrimental to cells for extreme oxygen consumption^[38]^.

## Dysregulation of mitochondrial biogenesis in aging-related diseases

Aging brings a general decline in physiological functions. This process is featured by mitochondrial decay and decrease of oxidative phosphorylation (OXPHOS) capacity, along with changes in mitochondrial morphology and mitochondrial content (number and protein levels)^[51]^. Defective mitochondria, a hallmark of cellular aging, include malfunction of mitochondrial biogenesis, abnormal mitochondrial dynamics and trafficking, aberrant autophagy function, and transcriptional dysregulation^[52–53]^. The ability of mitochondrial biogenesis is in slow and progressive decline with age. Hence, mitochondrial biogenesis has been viewed as a target for delaying aging and extending lifespan^[35,53–54]^. Mitochondrial biogenesis, a self-renewal route, aims to generate new mitochondria from the existing one, in order to meet energy requirements and maintain the dynamic circulation of mitochondria through collaborating with mitochondrial autophagy^[55]^. Therefore, mitochondrial biogenesis plays an important role in maintaining homeostasis of the mitochondrial mass and function, malfunction of which is associated with aging, neurodegenerative diseases, metabolic diseases, and cancers^[56]^.

Tremendous efforts have been made to discover the role of mitochondrial biogenesis-related proteins and genes under pathological condition. Accumulative literature has reported that mtDNA deletion, mutation and damage result in mitochondrial dysfunction among different tissues or within the same tissue, eventually responsible for aging and age-related neurodegenerative diseases^[57–58]^. The accumulation of mtDNA mutations triggers neuronal loss in the substantia nigra of patients with PD or rat PD models^[59–60]^, and negatively affects neuronal mitochondrial energy and synapse in the frontal cortex and hippocampus of AD patients^[61]^. Substantial evidence similarly shows that mtDNA damage is closely related to Huntington's disease (HD)^[53,62–63]^.

In another way, enhancement of mtDNA repair alleviates lung endothelial barrier dysfunction induced by donation after circulatory death related ischemia-reperfusion injury^[64]^. mtDNA copy number (mtDNAcn), regulated by transcriptional and translational factors, represents the mitochondrial abundance within a cell and varies with cellular energy requirement^[65]^. Alternations in mtDNAcn are associated with both increased and decreased disease burdens^[51]^. Some researchers hold the view that the upregulation of mtDNAcn due to overexpression of TFAM prolongs lifespan in the mouse suffering from mitochondrial diseases or myocardial infarction^[66–67]^. On the contrary, increased mtDNAcn negatively impacts the replication and transcription of mitochondrial proteins, accompanied by nucleoid enlargement^[68]^. These changes harm mitochondrial functions.

In brief, damaged mitochondrial biogenesis contributes to an accumulation of old or dysfunctional mitochondria and the progression of various diseases.

## Interaction between SIRT3 and mitochondrial biogenesis in aging-related diseases

**Figure 2:**
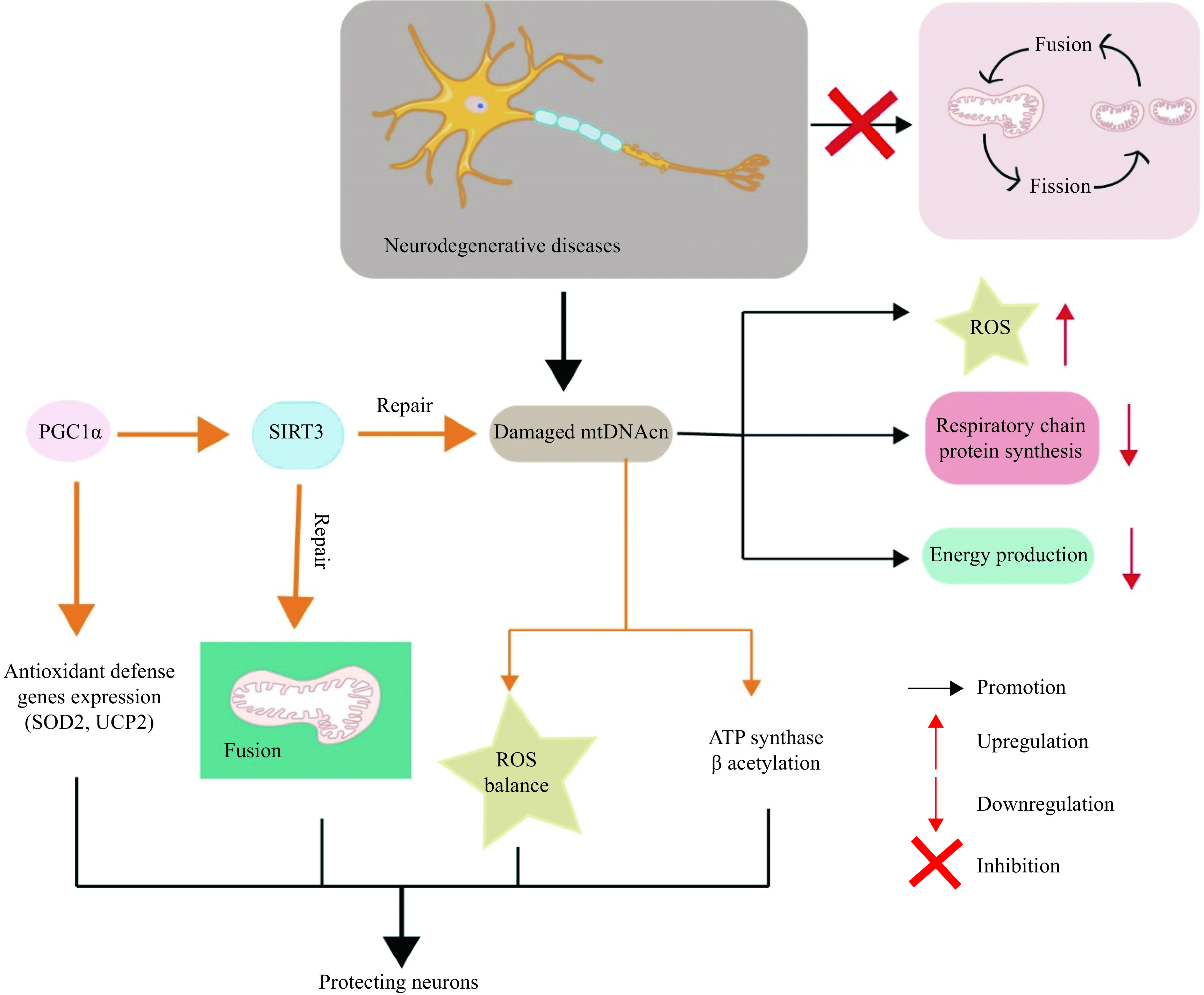
Interaction between SIRT3 and PGC1α in neurodegenerative diseases.

**Figure 3:**
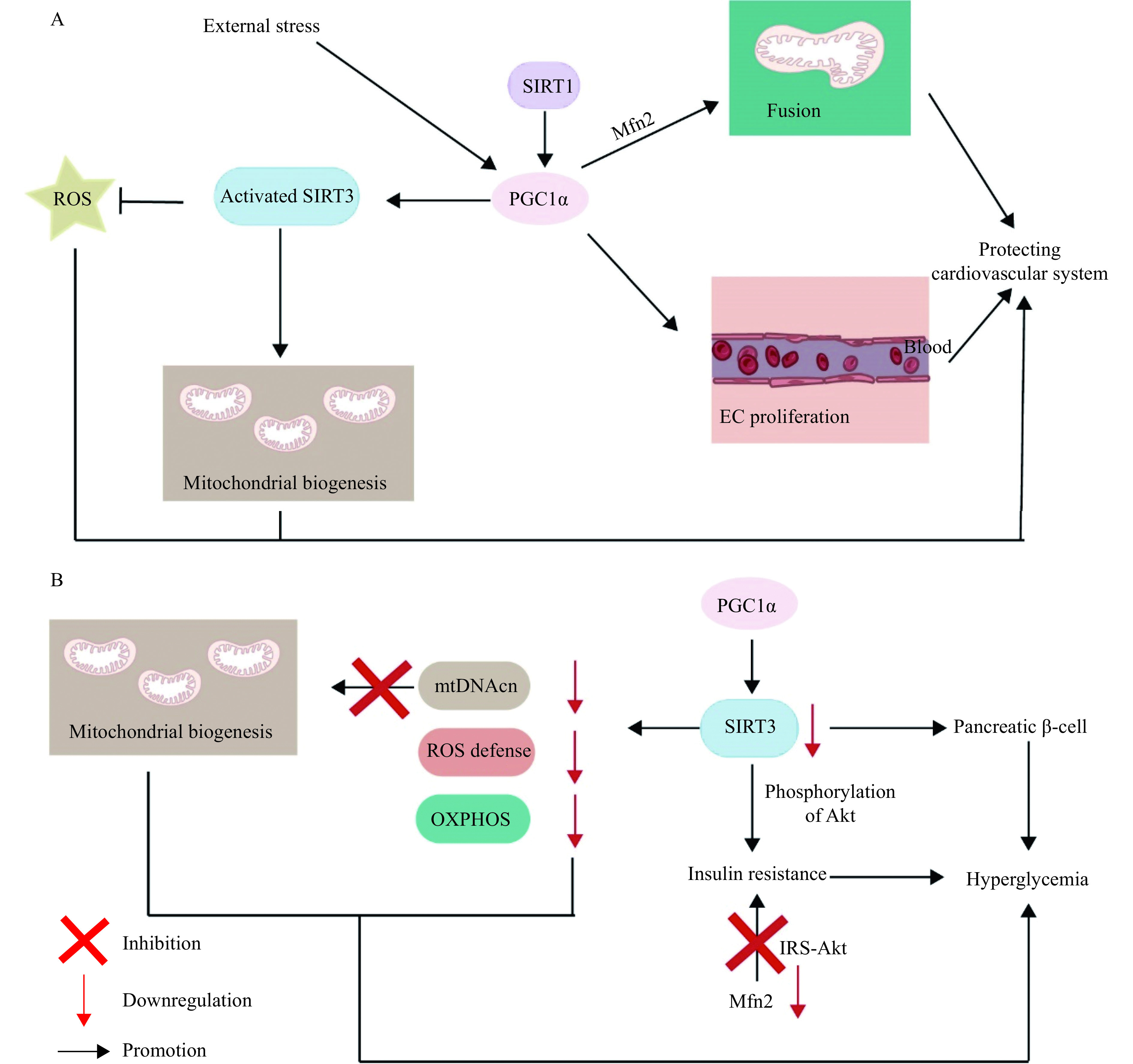
Interaction between SIRT3 and PGC1α in cardiovascular diseases and type 2 diabetes mellitus.

At the subcellular level, SIRT3 mainly localizes in mitochondria and deacetylates many mitochondrial metabolic proteins, and such observation was identified by mass spectrometry^[69]^. Likewise, mitochondrial metabolism such as fatty acid metabolism, glycolysis, and the tricarboxylic acid cycle is rich in acetylated proteins. SIRT3 plays a major role in sustaining mitochondrial bioenergetics. Thus, the pathway of SIRT3 and mitochondrial biogenesis is activated to maintain normal mitochondrial function and protect cell from death under pathological condition. In this part, we reviewed mitochondrial biogenesis with SIRT3 in three major aging-related diseases: cardiovascular diseases, neurodegenerative diseases, and T2DM. Additionally, the role of mitochondrial dynamics in the course of these diseases was summarized (***Fig. 2*** and ***Fig. 3***).

## Neurodegenerative diseases

Neurodegenerative diseases include AD, PD, HD, and amyotrophic lateral sclerosis (ALS). Featured by high-energy demand, neurons are vulnerable to oxidative stressors. Thus, the decrease of ATP level will result in neuronal death in patients with neurodegenerative diseases^[70]^. Mitochondrial malfunction and oxidative damage are pivotal contributors to the development of neurodegenerative diseases^[71–73]^.

The mtDNA or nuclear DNA mutations are reported to cause diseases related to neuronal degeneration^[71]^. The mtDNA replication machinery is more error-prone than that of genomic DNA, because the mtDNA polymerase lacks the function of proofreading^[34,74]^. Besides, DNA repair system is less reliable. The mtDNA damage can impair respiratory chain protein synthesis, reduce the efficiency of energy production and overproduce ROS. An increase in ROS impairs mtDNA in the same manner and misfolds proteins, *vice versus*^[75]^. These processes are related to mitochondrial dysfunction, eventually accelerating the development of diseases such as HD, AD, ALS, and hypertension^[72,74]^. However, SIRT3 possesses the ability to repair mtDNA damage, preserve mitochondrial function and protect apoptotic cell against death through deacetylating a variety of protein targets. These proteins consist of endonuclease Ⅷ-like 1, endonuclease Ⅷ-like 1 (NEIL2), 8-oxoguanine-DNA glycosylase 1 (OGG1)^[76]^, mutY DNA glycosylase (MUTYH), apurinic/apyrimidinic endonuclease (APE1), and DNA ligase 3 (LIG3)^[77]^, which modulate the activity of mtDNA base excision repair (BER), consequently taking charge of removing damaged bases. A large body of findings support that mtDNA repair machinery is impaired in neurodegenerative disorders^[78–80]^. In *Caenorhabditis* *elegans* PD models, incomplete BER results in genomic stress, promoting neuronal loss and further driving pathological process of age-related neurodegeneration^[81]^. Evidence supporting a critical role for DNA repair deficiencies in AD demonstrated that compromised DNA repair is a driving force of neuronal dysfunction and loss, because of damaged mitophagy, metabolic disturbance, and energy deprivation^[82]^. Research in ALS patients confirmed the perspective that altered mtDNA (mtDNA mutations and deletions) was observed in spinal neurons of ALS, driving synaptic dysfunction and facilitating motor neuron degeneration^[83]^. Altogether, studies concerning mtDNA alternations or ineffective BER demonstrate they have tight connection with neurodegenerative diseases.

SIRT3 and PGC-1α play a neuroprotective role in ALS model through preventing mitochondrial fragmentation and neuronal cell apoptosis^[84]^. SIRT3 is beneficial to maintaining ROS balance between production and clearance, overexpression of which suppresses ROS accumulation. PGC1α, strongly detected in tissues with high requirement for energy, not only induces antioxidant defense gene expression such as SOD2 and uncoupling protein 2 to stop oxidative damage and mitochondrial destruction^[85]^, but also modulates SIRT3 expression to achieve ROS homeostasis. In mutant SOD1 mice, motor neurons were found to develop degeneration due to aberrated mitochondria aggregation in neuronal axons and dendrites^[72]^. Knockout of SIRT3 suppresses antioxidant gene expression, leading to mitochondrial disequilibrium of antioxidant defense controlled by PGC1α^[84]^. Additionally, research in neuro-2a cell and SIRT3-knockout mice demonstrated that loss of SIRT3 had a deleterious effect on SOD2 and ATP synthase β acetylation levels targeting functional sites (SOD2-K130 and ATP synthase b-K485), critical for the regulation of ROS and ATP levels^[86]^. ROS aggregation and reduction in ATP are responsible for neuronal death in PD mice. Another insight is that PGC1a is a regulator of SIRT3 through interacting with ERRα to protect against dopaminergic neuronal death, a hallmark of PD^[86]^.

The imbalance of mitochondrial dynamics is a key component in the pathogenesis of neurodegenerative diseases such as PD and AD^[87–88]^. In PD, α-syn aggregation in the neuron is capable to perturb the cycle of mitochondrial fusion and fission because of its deleterious effect on mitochondrial associated proteins including mitofusin 1 and 2 (Mfn1/2), DRP1, and MFF, correlative with an increase in aberrant mitochondria and inefficiency of neuronal signaling^[89–90]^. Mitophagy, functioning as degrading damaged mitochondria, is also under the adverse influence of α-syn in PD^[88]^. Interestingly, SIRT3 depends on optic atrophy 1 (OPA1), a mitochondrial fusion regulator, to maintain normal mitochondrial dynamics, ultimately preventing cell apoptosis *via* the effect on cytochrome c translocation and mitochondrial respiratory efficacy^[91]^. Recently, research in the HD model reveals that upregulated SIRT3 contributes to mitochondrial fusion, rather than fission, leading to remodeled mitochondrial function, dynamics, and distribution in neural cells, further exerting neuroprotective effect. Decline in DRP1 and Fis1 levels caused by SIRT3 suppresses mitochondrial fission and biogenesis, and it means that mitochondrial mass isn't affected in HD^[92]^. Overall, SIRT3 has certain impacts on neurodegenerative diseases through the modulation of mitochondrial dynamics.

## Cardiovascular diseases

A small number of mitochondria exist in cardiomyocytes, one of the highest energy consuming cell types^[93–94]^. Not surprisingly, mitochondria in the function of vascular endothelial cells (VECs) as a metabolic signaling regulator triggering cell proliferation or apoptosis instead of energy supply^[93]^. Aberrant mitochondrial function is a contributing factor to endothelial dysfunction, responsible for cardiovascular diseases such as cardiac hypertrophy and atherosclerosis.

SIRT3 plays a protective role in myocardial cells *via* different signaling pathways to promote mitochondrial biogenesis. SIRT3 protects cardiomyocytes from ischemia reperfusion injury by preventing mitochondria mediated apoptosis, and this effect has been related to the activation of the AMPK pathway^[15]^. One explanation is that AMPK, an energy sensor of cells, drives mitochondrial biogenesis machinery in response to energy demand^[40]^. This viewpoint was supported by research in H9c2 cell, overexpression of SIRT3 induced corresponding changes in expression of mtDNA encoded genes, SOD2 expression and activity through the AMPKα-PGC1α axis, contributing to mitochondrial biogenesis and protecting myocardial cells^[95]^. Another function of PGC1α in VECs is to modulate several antioxidant enzymes, strengthening ROS defenses^[96]^. Furthermore, PGC1α controls some genes expression related to fatty acid oxidation, the tricarboxylic acid cycle, electron transport chain, and oxidative phosphorylation^[97]^, promoting vascular endothelial growth factor (VEGF) expression^[92]^ and protects against apoptosis *via* the effect of 15-hydroxyeicosatetraenoic acid^[98]^. This process mentioned above accelerates VEC proliferation and activates vessel sprouting in response to environmental stimulus (*e.g.*, CR and hypoxia)^[93]^.

Respiratory chain generates amounts of cellular energy, along with free radicals and ROS as by-products. Minimal ROS are conducive to signal transduction in physiological surroundings, whereas elevated ROS damages the adjacent cell structures, and alters DNA, proteins, and other molecules, further causing cell death^[34]^. It's well documented that excessive mitochondrial ROS give rise to VEC dysfunction, leading to formation of atherogenesis and cardiac hypertrophy^[34,74]^. Mitochondria-targeted esculetin-induced SIRT3, not SIRT1 overexpression, a new lipoxygenase inhibitor, protects endothelial cells from death through AMPK-mediated nitric oxide pathway, thus attenuating plaque formation^[99]^. Another perspective is that SIRT1 and SIRT3 cooperate to drive the antiaging effects under CR condition^[100]^. Increased expression of SIRT1 deacetylates and activates PGC1α in response to nutrient limitation, which further reduces ROS production, and promotes antioxidant environment through coactivating transcription of SIRT3^[100–101]^. This relationship is beneficial for longevity because of elevated mitochondrial biogenesis and ROS detoxification.

Therefore, mitochondrial dynamics play a critical role in VECs. Fusion proteins such as mfn1, mfn2, and OPA1 are associated with VEGF-mediated angiogenesis and angiogenic function^[102–103]^. The Mfn2 unregulated by PGC1α and PGC1β can promote mitochondrial fusion^[104]^. After knocking out the mfn2 gene in the heart of the mouse, calcium (Ca2^+^) fails to transfer from sarcoplasmic reticulum to mitochondria, followed by disrupted Ca2^+^ signaling, and diminished cardiac contractility function^[105]^. Evidence indicates that lacking cardiac-specific mfn2 undermines cellular autophagy and impairs mitochondrial network function, ultimately leading to aberrant left ventricular function^[106]^.

## Type 2 diabetes mellitus

T2DM is featured by deficient mitochondrial function and ROS. SIRT3 deacetylates and modifies several mitochondrial protease activities to manage mitochondrial functions and maintain redox homeostasis. The reduction of SIRT3 function contributes to the development of insulin resistance (IR), and hallmark of the pathogenesis of T2DM^[107–108]^. Evidence from experiment in cultured human endothelial cells demonstrated that SIRT3 deficiency is implicated in endothelial IR because of a drop in phosphorylation of Akt and endothelial nitric oxide synthase^[109]^. Similarly, overexpression of SIRT3 ameliorates negative effect of pancreatic β-cell involving malfunction and apoptosis induced by palmitate^[110]^. β-cell dysfunction is related to dysregulation of insulin synthesis and insulin deficiency, further leading to hyperglycemia^[111–112]^. Hyperglycemia in turn promotes mitochondria fragmentation, and increases ROS production^[113]^.

In pre-diabetic model, the researcher found that PGC1α/SIRT3 axis of testis was disrupted, accompanied by mtDNAcn decline^[114]^. PGC1α and Sirt3 are implicated in various biological processes such as mitochondrial biogenesis, functional OXPHOS, and an active ROS defense system^[101]^. The impaired PGC1α/SIRT3 axis compromises respiratory capacity, and promotes oxidative stress. Additionally, mitochondrial dynamics is not only the link between impaired mitochondrial function and IR, but also implicated in the development of T2D^[115–117]^. Diabetes susceptible cybrid cell model has demonstrated that IR is a consequence of abnormal mitochondrial dynamics, because upregulating Mfn1/Mfn2 genes and depressing DRP1/Fis1 can remodel mitochondrial network, repairing the IR signaling^[118]^. According to some viewpoints, both insulin signaling and insulin sensitivity are manipulated by Mfn2. In Mfn2 KO mouse, glucose tolerance and IRS-Akt pathway were impaired, whereas hepatic glucose production was enhanced^[115]^. Reduced Mfn2 expression was also found in T2D patients' muscles.

## Conclusions and perspectives

This review summarizes the involvement of SIRT3 and mitochondrial biogenesis in aging-related diseases. Based on SIRT3 regulation of mitochondrial function, we reviewed that there are intersecting signaling pathways between SIRT3 and mitochondrial biogenesis such as AMPK-PGC1α axis, SIRT1-PGC1α axis, and PGC1α/SIRT3 axis in the development of aging-related diseases including neurodegenerative diseases^[19]^, cardiovascular diseases^[12]^ and T2DM^[114]^. Impaired mitochondrial dynamics such as mitochondrial fusion and fission participate in the process of aging-related disease progression as well. Notably, manipulation of SIRT3, mitochondrial biogenesis, and mitochondrial dynamics offers novel therapeutic options for these aging-related diseases. However, other aspects of mitochondrial dysfunction such as abnormal mitochondrial quality control, mitochondrial homeostasis imbalance, and mitophagy dysfunction also account for aging-related disease progression and development. Thus, future investigations on SIRT3-mediated mitochondrial function may aid in providing new pathways during the aging process.
